# Impact of concomitant COVID-19 on the outcome of patients with acute myocardial infarction undergoing coronary artery angiography

**DOI:** 10.3389/fcvm.2022.917250

**Published:** 2022-09-22

**Authors:** Michał Terlecki, Wiktoria Wojciechowska, Marek Klocek, Agnieszka Olszanecka, Adam Bednarski, Tomasz Drożdż, Christopher Pavlinec, Paweł Lis, Maciej Zając, Jakub Rusinek, Zbigniew Siudak, Stanisław Bartuś, Marek Rajzer

**Affiliations:** ^1^First Department of Cardiology, Interventional Electrocardiology and Arterial Hypertension, Jagiellonian University Medical College, Kraków, Poland; ^2^Student's Scientific Group in the First Department of Cardiology, Interventional Electrocardiology and Arterial Hypertension, Jagiellonian University Medical College, Kraków, Poland; ^3^Faculty of Medicine and Health Sciences, Jan Kochanowski University, Kielce, Poland; ^4^Second Department of Cardiology, Jagiellonian University Medical College, Kraków, Poland

**Keywords:** novel coronavirus, COVID-19, myocardial infarction, revascularization, comorbidity

## Abstract

**Background:**

The impact of COVID-19 on the outcome of patients with MI has not been studied widely. We aimed to evaluate the relationship between concomitant COVID-19 and the clinical course of patients admitted due to acute myocardial infarction (MI).

**Methods:**

There was a comparison of retrospective data between patients with MI who were qualified for coronary angiography with concomitant COVID-19 and control group of patients treated for MI in the preceding year before the onset of the pandemic. In-hospital clinical data and the incidence of death from any cause on 30 days were obtained.

**Results:**

Data of 39 MI patients with concomitant COVID-19 (COVID-19 MI) and 196 MI patients without COVID-19 in pre-pandemic era (non-COVID-19 MI) were assessed. Compared with non-COVID-19 MI, COVID-19 MI was in a more severe clinical state on admission (lower systolic blood pressure: 128.51 ± 19.76 vs. 141.11 ± 32.47 mmHg, *p* = 0.024), higher: respiratory rate [median (interquartile range), 16 (14–18) vs. 12 (12–14)/min, *p* < 0.001], GRACE score (178.50 ± 46.46 vs. 161.23 ± 49.74, *p* = 0.041), percentage of prolonged (>24 h) time since MI symptoms onset to coronary intervention (35.9 vs. 15.3%; *p* = 0.004), and cardiovascular drugs were prescribed less frequently (beta-blockers: 64.1 vs. 92.8%, *p* = 0.009), angiotensin-converting enzyme inhibitors/angiotensin receptor blockers: 61.5 vs. 81.1%, *p* < 0.001, statins: 71.8 vs. 94.4%, *p* < 0.001). Concomitant COVID-19 was associated with seven-fold increased risk of 30-day mortality (HR 7.117; 95% CI: 2.79–18.14; *p* < 0.001).

**Conclusion:**

Patients admitted due to MI with COVID-19 have an increased 30-day mortality. Efforts should be focused on infection prevention and implementation of optimal management to improve the outcomes in those patients.

## Introduction

Since the beginning of the global pandemic, over 425 million people worldwide and nearly 6 million people in Poland have contracted coronavirus disease (COVID-19) with associated reported deaths exceeding 6 million and 100 thousand worldwide and in Poland, respectively (1, 2). COVID-19 has now become one of the leading causes of death globally, with death number comparable to those from cardiovascular disease (CVD) or cancer (1, 2). Although there is currently a declining trend in infection rates, it is expected that the severe acute respiratory syndrome coronavirus 2 (SARS-CoV-2) infection will likely remain at least as a dangerous, periodically recurring disease at an endemic level.

The pandemic has significantly impacted the behavior of patients suffering from myocardial infarction (MI). Due to increased levels of anxiety associated with interactions with healthcare workers as well at least partially limited access to in- and out-patient setting, an overall decrease in the number of invasive procedures and number of patients admitted due to ST elevation myocardial infarction (STEMI) and an increased amount of time from the first symptoms of MI to intervention have been observed in comparison with the pre-pandemic period (3–5).

Furthermore, an increasing amount of data suggests that during COVID-19, not only is the respiratory system involved but data also demonstrate that the cardiovascular system may be affected which can lead to myocardial injury. These patients tend to have a significantly worse prognosis than those without the myocardial injury (6–8). So far, it has been proven that the inflammatory and immune response due to viral infection has also had a role in the pathogenesis of an acute MI (9, 10). However, there are only a few studies that have assessed the impact of the COVID-19 infection on the outcome in the setting of patients with acute MI requiring revascularization (11, 12).

Even though it intuitively appears to be obvious that the patients with signs of a viral infection and MI even when optimally treated [including percutaneous coronary interventions (PCIs)] have a worse prognosis than patients with MI but without a viral infection, there is still a profound need to quantify this difference in the form of a mortality risk which up to this point has not been quantified. This, in turn, will allow for the implementation of adequate strategies aiming to reduce the risk of an undesirable prognosis in patients suffering from MI complicated with a concomitant COVID-19. Moreover, there is less to no data about the differences in the clinical course, comorbidities, and other factors influencing the outcome in patients with myocardial infarction depending on the presence/absence of COVID-19. Thus, we decided to compare the groups of patients admitted to our hospital due to MI and qualified for coronary angiography with concomitant SARS-CoV-2 infection against a control group consisting of patients treated for MI in our hospital in the preceding year before the onset of the pandemic.

## Materials and methods

We retrospectively studied the medical records of all consecutive patients who were admitted due to MI with concomitant SARS-CoV-2 infection to the University Hospital in Krakow between 6 March 2020 and 15 May 2021. In this described period, all patients admitted to our hospital, including those with MI on admission were diagnosed with SARS-CoV-2 infection according to the WHO and Polish guidelines using the reverse transcription polymerase chain reaction (RT-PCR) method (rhino-oropharyngeal swab positivity for SARS-CoV-2 RNA) (13–15). Patients with COVID-19 were treated according to the treatment algorithm recommended by the Polish Association of Epidemiologists and Infectiologists (13, 14). All patients in our study were diagnosed with MI and received the standard medical therapy according to the European Society of Cardiology (ESC) guidelines, and all were ultimately qualified for coronary angiography (16, 17).

For the control group, we retrospectively studied the medical records of all consecutive patients who were admitted due to MI and qualified for coronary angiography to the University Hospital in Krakow between the period of 15 April 2019 and 15 September 2019. The time period for the non-COVID-19 MI group of patients chosen for analysis was selected to minimize the possible impact of other viral infections on the clinical course of MI (in our country, a peak incidence of respiratory viral infections has regularly been noticed between the months of January–March each epidemic season and nearly no incidences of these aforementioned infections during the mid-Spring to Summer seasons) (18). Additionally, this enabled us to avoid the possibility of inclusion in control group patients with undiagnosed SARS-CoV-2 infection or inclusion of patients with MI which occurred after SARS-CoV-2 infection (either diagnosed or undiagnosed) which could have had an impact on the outcome during MI. Cardiovascular risk factors and cardiovascular diseases were identified based on the previous medical history of diagnosis and/or treatment and defined according to the current ESC guidelines (19). All clinical data including demographics, medical history, inpatient clinical course, laboratory results, treatments, and in-hospital outcomes were obtained from the electronic medical records used by the University Hospital in Krakow. The estimated glomerular filtration rate (eGFR) was calculated from the Modification of Diet in Renal Disease (MDRD) formula (20). Heart rate, arterial blood pressure, Killip class, and Global Registry of Acute Coronary Events (GRACE) risk score were assessed in all patients based on their clinical condition (21). Thrombolysis in myocardial infarction (TIMI) coronary flow grade scores was evaluated before and after PCI (22). The primary percutaneous coronary intervention was defined as the strategy of taking a patient with MI directly to the cardiac catheterization laboratory to undergo mechanical revascularization. Transthoracic two-dimensional echocardiography was performed in patients during the admission to the Cardiology Department to measure left ventricular ejection fraction (LVEF). Based on the data obtained from the Universal Electronic System for Registration of the Population in Poland, the occurrence of death from any cause at 30 days was evaluated for all study participants. Our study was an observational retrospective analysis of anonymized electronic medical records of patients hospitalized in our hospital. The study was conducted according to the guidelines of the Declaration of Helsinki and approved by the Bioethics Committee of the Jagiellonian University (no. 1072.6120.278.2020 and no. 1072.6120.333.2020).

### Statistical analysis

Categorical variables were presented as numbers and percentages. Continuous variables were expressed as means and standard deviation (SD) or medians and interquartile range (IQR). Normality was assessed by the Shapiro–Wilk test. We divided the study population into two groups according to their diagnosis of COVID-19. Differences between groups were compared using the Student's or Welch's *t*-test depending on the equality of variances for normally distributed variables. The Mann–Whitney U test was used for non-normally distributed continuous variables. Cox-proportional hazards models were fit to determine the adjusted associations between cofounders (including COVID-19 status) and mortality. Variables that were associated with the occurrence of 30-day mortality with a significance level of *p* < 0.2 in the bivariable models as well as other variables judged to be of clinical importance were selected for possible inclusion in the multivariable logistic regression model to predict the occurrence of the outcome. Adjusted hazard ratios (HRs), along with 95% confidence intervals (CIs), were computed for all covariates. The proportional hazards model assumptions were checked using the Schoenfeld test and graphical diagnostics. Furthermore, to analyze event-free survival in 30-day follow-up after hospital admission due to MI, Kaplan–Meier curves were drawn for all patients stratified by COVID-19 status. In all analyses, a *p*-value of 0.05 or less was considered statistically significant. The statistical analysis was performed with the IBM SPSS 24.0 software package, STATA software, version 15 and R Core Team (2020).

## Results

### Study population and clinical characteristics

A total of 235 patients [94 women (40.0%)] with MI were reviewed. The mean age ± standard deviation (SD) was 68.46 ± 12.21 years. There were 79 (33.61%) patients with STEMI and 156 (66.38%) with no ST elevation myocardial infarction (NSTEMI). Arterial hypertension (82.55%) and diabetes (41.28%) were the predominant coexisting diseases. There were 58 patients (24.68%) with Killip class 3 or 4. Multivessel disease (MVD) was found in 85 subjects (36.17%). Median (interquartile range) time from the onset of symptoms to coronary angiography was 480.9 (240–1,200) min. Half of the study group was qualified for PCI (124 patients, 52.77%). Among patients who underwent PCI, STEMI was diagnosed in 44 patients (35.5%), left main coronary artery (LMCA) was infarct-related artery (IRA) in 3 patients (2.4%), and total occlusion of an IRA was seen in 36 patients (29.0%). TIMI 3 after PCI was achieved in 113 patients (93.4%).

There were 39 patients with MI and concomitant COVID-19 (COVID-19 MI group) and 196 patients with MI and without COVID-19 (non-COVID-19 MI group). There were no significant differences concerning age, gender, body mass index (BMI), and comorbidities (i.e., arterial hypertension, heart failure, chronic kidney disease, diabetes, atrial fibrillation, chronic obstructive pulmonary disease, history of previous myocardial infarction, and history of previous coronary artery bypass graft) observed between study groups (Table 1).

**Table 1:** Basic characteristics of participants.

**Characteristics**	**Non-COVID-19 MI**	**COVID-19 MI**	***p* value^$^**
	***N* = 196**	***N* = 39**	
	**(83.4%)**	**(16.6%)**	
Age, years, mean (SD)	68.03 (12.31)	70.64 (11.60)	0.233
Female sex, *n* (%)	79 (40.3)	15 (38.5)	0.489
BMI*, kg/m^2^, mean (SD)	26.66 (5.01)	27.01 (3.90)	0.324
**Pre-existing conditions**, ***n*** **(%)**			
Arterial hypertension	161 (82.1)	33 (84.6)	0.458
Diabetes mellitus	84 (42.9)	13 (33.3)	0.178
History of previous MI	52 (26.5)	12 (30.8)	0.358
History of CABG	7 (3.6)	3 (7.7)	0.220
Heart failure	55 (28.1)	11 (28.2)	0.563
Atrial fibrillation	28 (14.3)	10 (25.6)	0.069
Malignant disease	14 (7.1)	3 (7.7)	0.560
COPD	4 (2.0)	2 (5.1)	0.261
Chronic kidney disease	22 (11.2)	5 (12.8)	0.476

Data are presented as mean (SD), median (Q1–Q3), or number (%).

*Data available for 43 patients (35 for non-COVID-19 MI and 8 for COVID-19 MI).

^*$*^For between non-COVID-19 MI group and COVID-19 MI group difference.

BMI, body mass index; CABG, coronary artery bypass graft, COPD, chronic obstructive pulmonary disease; COVID-19, coronavirus disease 2019; MI, myocardial infarction.

### In-hospital course, angiographic findings, drugs therapy, and predictors of 30-day mortality

Patients with COVID-19 MI presented a more severe clinical state on admission (assessed by a lower systolic blood pressure, higher respiratory rate, higher GRACE score) in comparison with the non-COVID-19 MI group; however, there were no significant differences in the frequency of STEMI/NSTEMI, admission values of high sensitivity cardiac troponin (hs cTn), N-terminal prohormone of brain natriuretic peptide (NT-proBNP), and LVEF between groups (Table 2). The frequency rate of PCI and non-obstructive coronary artery disease were similar between study groups, and no significant differences were found in the IRA, the percentage of reaching TIMI 3 after PCI, the frequency rate of primary PCI or MVD, and the amount of contrast used during coronary invasive procedure. In patients with COVID-19 MI (in comparison with patients with non-COVID-19 MI), there was a significantly higher percentage of patients with prolonged time (>24 h) from the initial onset of MI symptoms to coronary intervention. Patients with MI and COVID-19 had significantly higher levels of peak NT-proBNP, but there were no differences in peak hs cTn and peak creatinine during the hospital stay when compared to patients with non-COVID-19 MI. In comparison with patients with non-COVID-19 MI, COVID-19 MI subjects received less frequently cardiovascular drugs (beta-blockers, angiotensin-converting enzyme inhibitors/angiotensin receptor blockers, and statins). Even though there were no differences between the study groups in the frequency rate of need for mechanical ventilation and catecholamine use during a hospital stay, there was a significantly higher 30-day mortality rate in patients with MI and COVID-19 (Table 3).

**Table 2:** Clinical characteristics on admission among non-COVID-19 MI and COVID-19 MI group.

**Parameters on admission**	**Non-COVID-19 MI**	**COVID-19 MI**	***p* value^$^**
	***n* = 196**	***n* = 39**	
	**(83.4%)**	**(16.6%)**	
SBP*, mmHg, mean (SD)	141.11 (32.47)	128.51 (19.76)	0.024
DBP*, mmHg, mean (SD)	81.26 (17.92)	79.16 (13.37)	0.499
Heart rate*, /min, mean (SD)	80.23 (18.04)	84.27 (19.58)	0.221
Respiratory rate*, /min, median (IQR)	12 (12; 14)	16 (14; 18)	<0.001
GRACE score*, mean (SD)	161.23 (49.74)	178.50 (46.46)	0.041
Killip 4 class, n (%)	28 (14.3)	5 (12.8)	0.521
STEMI, n (%)	62 (31.6)	17 (43.6)	0.105
Ejection fraction*, mean (SD)	45.25 (14.52)	43.00 (15.19)	0.400
NT-proBNP*, pg/ml, median (IQR)	2,866.00 (767.00; 8,570.50)	6,192.00 (1,071.00; 18,263.00)	0.076
hs cTn*, ng/ml, median (IQR)	2,449.55 (560.60; 11,440.29)	7,503.89 (1,154.93; 21,844.29)	0.063
Creatinine*, μmol/l, median (IQR)	87.00 (70.00; 114.00)	112.00 (71.45; 155.00)	0.022

Data are presented as mean (SD), median (Q1–Q3), or number (%).

^*$*^for the difference between non-COVID-19 MI group and COVID-19 MI group.

*Data available in: 229 patients for SBP and DBP; 152 patients for NT-pro BNP; 235 patients for hs cTn—high-sensitivity cardiac troponin; 224 patients for creatinine; 235 patients for heart rate; 228 patients for respiratory rate; 235 patients for GRACE score; 231 patients for EF.

COVID-19, coronavirus disease 2019; DBP, diastolic blood pressure; GRACE, Global Registry of Acute Coronary Events; LVEF, left ventricular ejection fraction; hs cTn, high-sensitivity cardiac troponin; NT-proBNP, N-terminal prohormone of brain natriuretic peptide; SBP, systolic blood pressure; STEMI, ST-elevation myocardial infarction.

**Table 3:** Angiography results, in-hospital drug therapy and patient's outcome among non-COVID-19 MI and COVID-19 MI group.

**Parameter**	**Non-COVID-19 MI**	**COVID-19 MI**	***p*-value**
	***N* = 196**	***N* = 39**	
	**(83.4%)**	**(16.6%)**	
Multivessel disease, *n* (%)	66 (33.7)	19 (48.7)	0.056
Time from onset of symptoms to cardiac intervention >24 h, *n* (%)	30 (15.3)	14 (35.9)	0.004
PCI, *n* (%)	105 (53.6)	19 (48.7)	0.352
Non-obstructive coronary arteries, *n* (%)	39 (19.9)	10 (25.6)	0.272
Primary PCI, *n* (%)*	92 (87.6)	18 (94.7)	0.328
Acute total occlusion of IRA, *n* (%)*	32 (30.5)	4 (21.1)	0.296
STEMI, *n* (%)*	36 (34.3)	8 (42.1)	0.341
Infarct related artery*
LAD, *n* (%)*	43 (41.0)	8 (42.1)	0.559
LMCA, *n* (%)*	3 (2.9)	0 (0.0)	0.604
Cx, *n* (%)*	29 (27.6)	6 (31.6)	0.459
RCA, *n* (%)*	28 (26.7)	5 (26.3)	0.610
TIMI 3 after PCI, *n* (%)*	97 (95.1)	16 (84.2)	0.110
Time from onset of symptoms to PCI > 24 hours, *n* (%)*	17 (16.19)	8 (42.11)	**0.010**
Contrast, ml, median (IQR)*	200 (150; 250)	220 (200; 300)	0.408
Peak hs cTn, ng/ml, median (IQR)	9,559.71 (2,542.90; 25,000.00)	11,899.43 (2,764.94; 25,000.10)	0.730
Peak NTproBNP, pg/ml, median (IQR)	2,984.00 (796.00; 10,246.00)	6,329.00 (1,733.00; 18,263.00)	**0.034**
Peak creatinine, μmol/l, median (IQR)	103.00 (80.00; 136.00)	134.00 (86.05; 189.00)	0.071
ACEI/ARB, *n* (%)	159 (81.1)	24 (61.5)	**0.009**
Beta blockers, *n* (%)	180 (91.8)	25 (64.1)	<**0.001**
Statins, *n* (%)	185 (94.4)	28 (71.8)	<**0.001**
Catecholamines, *n* (%)	28 (14.3)	5 (12.8)	0.521
Mechanical ventilation, *n* (%)	23 (11.7)	5 (16.6)	0.513
Death in 30-day follow-up	17 (9.4)	12 (38.7)	<**0.001**

*Percentages and median calculated only among patients who underwent PCI.

ACEI, angiotensin-converting enzyme inhibitors; ARB, angiotensin receptor blockers; COVID-19, coronavirus disease 2019; Cx, left circumflex artery; hs cTn, high-sensitivity cardiac troponin; IRA, infarct-related artery; IQR, interquartile range; LAD, left anterior descending artery; LMCA, left main coronary artery; MI, myocardial infarction; NT-proBNP, N-terminal prohormone of brain natriuretic peptide; OMT, optimal medical treatment; RCA, right coronary artery; STEMI, ST-elevation myocardial infarction; TIMI, thrombolysis in myocardial Infarction; PCI, percutaneous coronary intervention.

**Figure 1:**
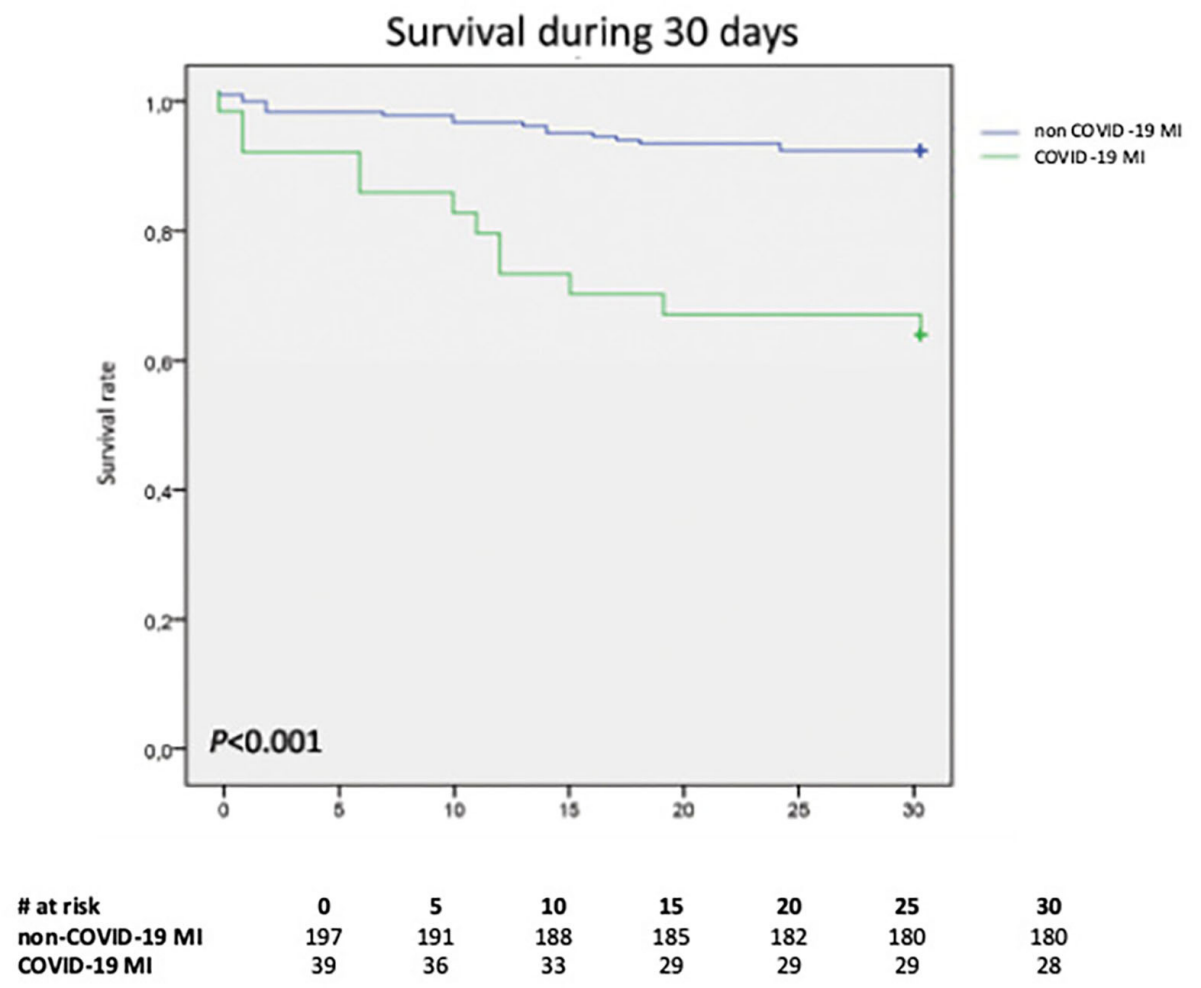
The Kaplan–Meier curve displaying proportional 30-day mortality from any cause stratified by COVID-19 status; *p* < 0.001. COVID-19, coronavirus disease 2019; MI, myocardial infarction.

The Kaplan–Meier curves in Figure 1 display that patients with non-COVID-MI had higher survival rate, than patients with COVID-19 MI based on a 30-day observation period (Figure 1).

**Figure 2:**
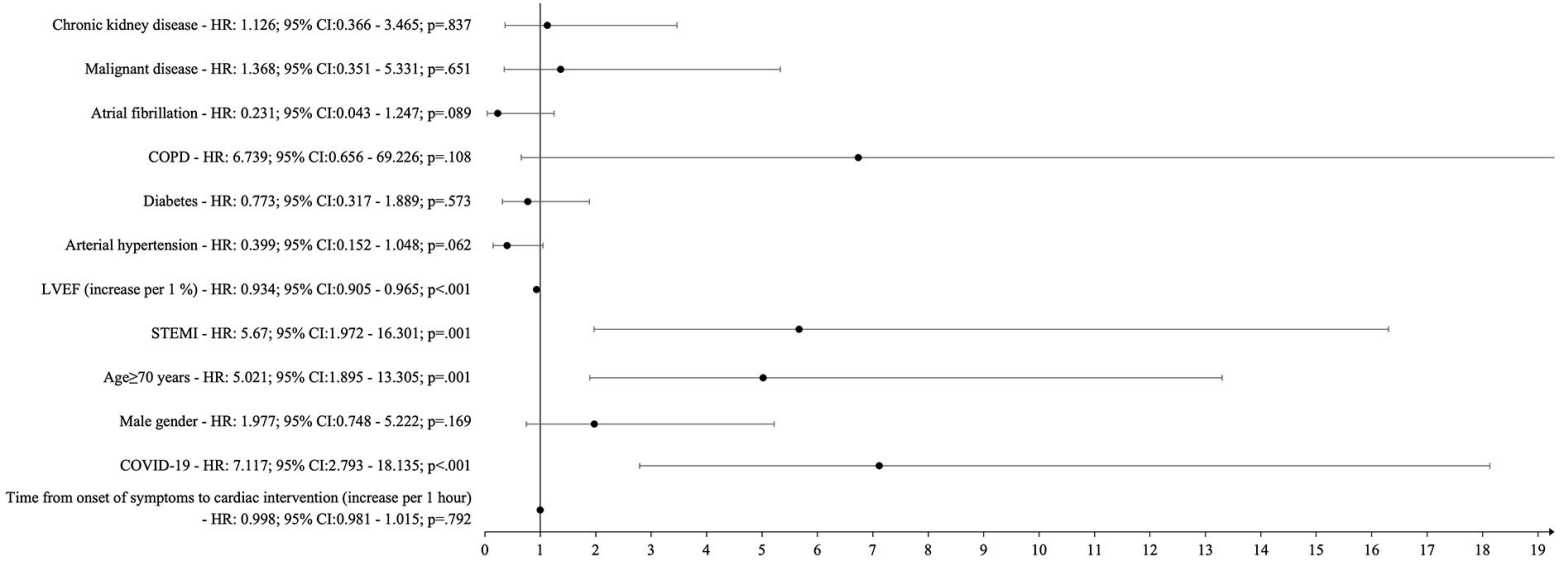
Cox regression analysis: independent predictors of 30-day mortality. CI, confidence Interval; COPD, chronic obstructive pulmonary disease; COVID-19, coronavirus disease 2019; LVEF, left ventricular ejection fraction; STEMI, ST-elevation myocardial infarction.

In the Cox-proportional hazards model advanced age, STEMI, reduced LVEF, and COVID-19 were associated with an increased risk of 30-day mortality (Figure 2).

## Discussion

The main finding of our study is that the patients who have suffered from both acute MI and have contracted the COVID-19 have a significantly higher 30-day risk of mortality compared to those patients with MI who did not have COVID-19. Despite no significant differences in demographics or comorbidities, patients with COVID-19 in our analysis had a worse clinical state at the admission, less frequently received guidelines-recommended medication and coronary intervention that was delayed.

Due to the relatively short observational period of pandemic, the comprehensive reports on the effect of concomitant COVID-19 on the diagnosis, treatment, and outcomes in patients with MI are still being carried out. In North American registry (NACMI), patients with STEMI and COVID-19 were compared to a control group of patients with STEMI treated 5 years before the pandemic (23). This enabled researchers to study both the impact of infection itself on the outcome and to analyze the effect of pandemic on the management of patients with MI (23). In NACMI registry, patients with COVID-positive STEMI were found to have more severe clinical condition before PCI (higher rates of cardiac arrest and cardiogenic shock) in comparison with control group (23). In this registry, the in-hospital mortality rate in COVID-19 group of patients was significantly higher (33%) than in the control group (4%) (23). Additionally, delayed coronary intervention was also observed in this registry among patients with COVID-19 (23). In contrast to our study, the NACMI registry included only patients with STEMI (23) but in another international registry of acute coronary syndromes in patients with COVID-19, Kite et al. (24) included patients with both STEMI and NSTEMI COVID-19-positive and highly suspicious for COVID-19 who underwent invasive coronary angiography and compared them with pre-COVID-19 cohort. It has been observed that in-hospital mortality in patients with COVID-19 was significantly higher than in control subjects in both STEMI and NSTEMI groups (reaching 22 and 6% in STEMI and NSTEMI, respectively) (24). Both the NACMI registry and study of Kite et al. did not assess the possible effect of the frequency of cardiac guidelines-recommended medication in patients with MI according to their COVID-19 status (23, 24). In our study, we included only confirmed by RT-PCR tests COVID-19 cases and additionally, the data about cardiac medication therapy were also assessed.

Both the findings from the above-mentioned registries (23, 24) and the results of our study prompt a deeper consideration of to what extent a COVID-19 itself, and to what pandemic-related side factors (delays in patients admission to hospital, logistical challenges related to health systems reorganization, etc.) influenced the higher mortality observed in our study among patients with MI and COVID-19 in comparison with pre-pandemic MI control group. It has been proven that patients with cardiovascular diseases including MI have limited cardiac, renal, and/or pulmonary reserve, making them more susceptible to complications arising from SARS-CoV-2 infection leading to a more severe clinical course (25, 26). In our study, patients with MI and COVID-19 had higher scores on the GRACE scale, lower systolic blood pressure values, and higher respiratory rates compared to patients with non-COVID-19 MI. Those observations may be due to the fact that COVID-19 does not solely affect the respiratory system but also often causes multi-organ failure that can present itself as myocardial injury or aggravation of kidney disease which negatively affects the prognosis of patients suffering from COVID-19 (27). Our observations of significantly different levels of heart and renal failure laboratory markers (higher baseline creatinine and peak NT-proBNP in patients with COVID-19 MI) between study groups support the thesis of cardiac and/or renal involvement in patients with COVID-19. The results of NACMI registry and study of Kite et al. also confirm both respiratory and cardiac involvement in patients with COVID-19 MI (23, 24). Kite et al. (24) reported that among patients with cardiogenic shock, one of the dominant cause of death (31% of cases) was respiratory (despite severe conditions due to cardiogenic shock). In NACMI registry, patients with COVID-19 were reported to have frequently pulmonary infiltrates on chest X-ray (23). Having in mind that it has been proven previously that viral infections (i.e., influenza, SARS, and MERS) have been proven to exacerbate MI (28, 29), we postulate that results of our study and above-mentioned registries confirm that COVID-19 should be considered as the dominant cause of increased mortality in this group of patients.

Since the beginning of pandemic in our hospital, over 5,000 patients with COVID-19 have been hospitalized and precise correlations between cardiovascular diseases and their prognosis have been thoroughly described in our other publication (30). We observed that significant number of patients with COVID-19 present with increased myocardial injury markers (more than 40% for hs cTn and more than 80% for NT-proBNP) which agrees with other published findings (31–34). This might suggest that the process of differentiating myocardial injury from MI in patients with concurrent COVID-19 remains challenging. Additionally, the diagnosis of MI with indications for coronary innervation might be time-consuming in those patients. This may also attribute to the significantly longer time delay since onset of symptoms to intervention. There are numerous studies confirming that time delay to treatment is a significant factor associated with an increased risk of heart failure and mortality in patients with MI (35–37). Scholz et al. (35) demonstrated that every 10-min treatment delay resulted in 3.31 additional deaths in 100 PCI-treated patients with STEMI patients with cardiogenic shock. In the study of Terkelsen et al. (36), system delay was an independent risk factor of increased 30-day mortality; however, it was confirmed only for STEMI of anterior wall. The delay in revascularization treatment in patients with MI and COVID-19 has also been reported (3, 23, 38). Our findings show that 35.9% of patients with MI and COVID-19 underwent the cardiovascular intervention after more than 24 h from the onset of symptoms; however, we did not confirm that time since onset of symptoms to coronary angiography was an independent factor of increased risk of 30-day mortality, which can be explained by the fact that in our study, in contrast to above-mentioned studies, we included patients with both STEMI and NSTEMI and there was a significant number of patients who did not require PCI. Additionally, we must acknowledge that the cause for the treatment delay for patients with COVID-19 MI is multifactorial (including patient's delayed presentation and healthcare re-organization during pandemic).

It has been suggested that MI with non-obstructive coronary artery (MINOCA) is being frequently observed in patients with COVID-19 (11, 23, 39). MINOCA is a heterogeneous group of disorders including Takotsubo syndrome, myocarditis, transient thrombosis, or type 2 MI which must be taken into consideration in diagnostic process in patients with MI and concomitant COVID-19. The incidence of MINOCA varied across the studies of patients with MI and COVID-19 from 26 up to 56% (11, 23, 39). In our study, the frequency rate of MINOCA reached 25% in COVID-19 group of patients, but there were no significant differences in comparison with patients with non-COVID-19 MI. We must admit that in our daily practice (including both pre-pandemic and pandemic periods), advanced non-invasive diagnostic tools such cardiac magnetic resonance imaging should be used more frequently to determine the underlying causes of myocardial infarction with non-obstructive coronary arteries and to reduce the number of coronary angiography not requiring PCI.

It is recommended that patients with MI should be treated with beta-blockers, angiotensin-converting enzyme inhibitors, or angiotensin-receptor blocker and statins as it has been proven that these drugs improve the prognosis of patients after MI (16, 17). In our research, the aforementioned drugs have been prescribed at a much lower rate in patients with MI and COVID-19 compared to patients with non-COVID-19 MI. Due to the retrospective character of our work, we can only speculate on the causes of this discrepancy. It should be assumed that there were clinical contraindications that may have influenced the decision to use these medications in the COVID-19 MI group. Such contradictions may include renal failure or tendencies for hypotension and might have had an impact on poor outcome.

When analyzing the mortality risk of patients with COVID-19 MI, we must take into consideration a possible contribution of pandemic-related collateral factors on final outcome (patients' need for self-isolation or fear of catching the infection, delays in patients' admission to hospital, logistical challenges related to health systems reorganization, etc.). High mortality risk of patients with COVID-19 observed in NACMI registry (23) in a study of Kite et al. (24) or reported by us shows sevenfold increased risk of 30-day mortality in patients with COVID-19 in comparison with pre-pandemic MI control group which may at least be partially explained by above-mentioned non-infectious factors. Thus, efforts toward the reduction of the mortality risk in this group of patients should be focused not only on the prevention of SARS-CoV-2 infection and implementation of COVID-19 effective treatment but also on the improvement in diagnosis of MI, optimization of both interventional and medical treatment, and efficient health system organization.

Our study has several limitations. First, the retrospective study design limits the ability to obtain complete data for patients' characteristics; second, we could not distinguish between type I (plaque rupture/erosion) and type 2 MI (supply demand mismatch alone). In our hospital, inflammatory markers were not typically drawn during routine blood testing for patients with MI before the COVID-19 pandemic; thus, we could not test the association between inflammatory marker levels and the prognosis in both groups of patients. It is also important to underline that in MI patients with COVID-19, GRACE score results should be interpreted with caution because there may be several factors that could have contributed to the altered heart rate or blood pressure in patients with COVID-19 (i.e., fever, hypovolemia, etc.). Additionally, it is worth to underline that pandemic has greatly impacted the healthcare system and modified the management strategies in patients with MI. The possible impact of pandemic itself (i.e., delay in hospital admission due to fear of COVID-19 infection, temporary lockdown, temporarily shifting resources to the treatment of only acute cases, shortage of ambulance transport, and shortage of staff) must be taken into consideration as a possible additional factors responsible for poor outcomes of COVID-19 MI group of patients in comparison with control group (patients with non-COVID-19 MI in pre-pandemic era).

## Conclusion

Patients admitted due to acute MI with COVID-19 have increased 30-day mortality in comparison with patients with MI in the pre-pandemic era. Efforts should be focused on the infection prevention and the implementation of optimal management to improve outcome in those patients.

## Data availability statement

The raw data supporting the conclusions of this article will be made available by the authors, without undue reservation.

## Ethics statement

The studies involving human participants were reviewed and approved by Bioethics Committee of the Jagiellonian University (No. 1072.6120.278.2020 and No. 1072.6120.333.2020). Written informed consent for participation was not required for this study in accordance with the national legislation and the institutional requirements.

## Author contributions

MT, WW, AB, TD, CP, PL, MZ, JR, and MR contributed to the conception and design or analysis and interpretation of data, or both. MT, WW, MK, AO, AB, TD, CP, PL, MZ, JR, ZS, SB, and MR drafted the manuscript or revised it critically for important intellectual content. All authors approved the submitted manuscript.

## Funding

This publication was supported by the National Center for Research and Development CRACoV-HHS project (Model of Multi-Specialist Hospital and Non-Hospital Care for Patients with SARS-CoV-2 Infection) through the initiative Support for specialist hospitals in fighting the spread of SARS-CoV-2 infection and in treating COVID-19 (Contract no. SZPITALE-JEDNOIMIENNE/18/2020). The research was implemented by a consortium of the University Hospital in Cracow and the Jagiellonian University Medical College.

## Conflict of interest

The authors declare that the research was conducted in the absence of any commercial or financial relationships that could be construed as a potential conflict of interest.

## Publisher's note

All claims expressed in this article are solely those of the authors and do not necessarily represent those of their affiliated organizations, or those of the publisher, the editors and the reviewers. Any product that may be evaluated in this article, or claim that may be made by its manufacturer, is not guaranteed or endorsed by the publisher.
